# Fuzhenghuayu Decoction ameliorates hepatic fibrosis by attenuating experimental sinusoidal capillarization and liver angiogenesis

**DOI:** 10.1038/s41598-019-54663-4

**Published:** 2019-12-10

**Authors:** Hong-liang Liu, Jing Lv, Zhi-min Zhao, An-ming Xiong, Ye Tan, Jeffrey S. Glenn, Yan-yan Tao, Hong-lei Weng, Cheng-hai Liu

**Affiliations:** 1 Institute of Liver Diseases, Shuguang Hospital affiliated to Shanghai University of Traditional Chinese Medicine, Shanghai, 201203 China; 2 Shanghai Key Laboratory of Traditional Chinese Clinical Medicine, Shanghai, 201203 China; 3 Key Laboratory of Liver and Kidney Diseases, Ministry of Education, Shanghai, 201203 China; 4 Division of Gastroenterology and Hepatology, Department of Medicine, Stanford University School of Medicine, Stanford, California United States; 5 Department of Medicine II, Section Molecular Hepatology, Medical Faculty Mannheim, Heidelberg University, Mannheim, Germany

**Keywords:** Liver fibrosis, Hepatic stellate cells

## Abstract

Fuzhenghuayu (FZHY) is a compound extracted from natural plants. Its anti-fibrotic effect has been confirmed in experimental and clinical studies. However, precise effects and underlying mechanisms of FZHY in liver angiogenesis largely remain understood. In this study, we investigated the effects of FZHY on sinusoidal capillarization and angiogenesis with mice challenged for Carbon tetrachloride (CCl_4_) and dimethylnitrosamine (DMN), *in vitro* human hepatic sinusoidal endothelial cells (HHSEC) and Human Umbilical Vein Endothelial Cell (HUVEC) 3D fibrin gel model. Besides its anti-fibrotic effect, FZHY ameliorated CCl_4_ and DMN-induced sinusoidal capillarization, angiogenesis and expression of angiogenesis-associated factors, i.e. CD31, VEGF, VEGF receptor II, phosphor-ERK and HIF-1α. Consistent with the findings based on animal models, inhibitory effects of FZHY on capillarization and angiogenesis were further confirmed in HHSEC and the HUVEC 3D fibrin gel model, respectively. These data suggest that FZHY ameliorates not only liver fibrosis but also vessel remodeling in experimental models. Therefore, FZHY might be a potentially useful drug to treat liver cirrhosis in clinical practice.

## Introduction

The pathophysiology of liver fibrosis is a complex biological process characterized as aberrant healing, excessive deposition of extracellular matrix proteins and increased angiogenesis^1–4^. Capillarization of liver sinusoidal endothelial cells (LSECs), also called dedifferentiation, occurs following liver injury. Sinusoidal capillarization and liver angiogenesis are two key events leading to liver cirrhosis^5^. Normal differentiated LSECs are characterized by the presence of fenestra and the absence of base membrane. Such a structure arrangement guarantees nutrition and molecule exchange between hepatocytes and sinusoidal blood. Maintenance of fenestra of LSECs requires signals from hepatic stellate cells (HSCs), the key cell source of extracellular matrix during liver fibrogenesis^6,7^. However, any hepatic injury-activated HSCs results in de-differentiation of LSECs, which is morphologically presented as fenestra loss, sinusoidal capillarization^8^. Sinusoidal capillarization is the structural basis of multiple pathophysiological alterations in liver diseases, e.g. liver dysfunction and hypoxia. Hypoxia causes liver angiogenesis^9^. Structural changes in the sinusoids including basement membrane deposition and loss of LSEC fenestra, in turn lead to impaired oxygen diffusion from the sinusoids to the parenchyma. Accumulation of HIFs, composed of HIF-1α, HIF-2α and HIF-1β, stimulate expression of VEGF and its receptors, and thus enhancing the hypoxia-induced angiogenesis^10–12^, and stimulating further HSC activation^13^.

Angiogenesis inside fibrotic septa connecting portal tracts and central veins is one of the key events underlying liver cirrhosis^14^. Such vascularized fibrous septa generate “a bridge too far” and result in parenchymal extinction and portal hypertension^15^. Pharmacologic intervention, e.g. utilizing receptor tyrosine kinase inhibitors Sorafenib (SORA) or Sunitinib, not only inhibit angiogenesis, but alsodecrease hepatic fibrosis^16–18^. Thus, inhibiting sinusoidal capillarization and vascularized fibrous septa are crucial for prevention of liver cirrhosis.

FZHY, a China Food & Drug Administration (CFDA)-approved Chinese medicinal formulation, has been used to treat HBV relevant liver fibrosis in China for decades^19,20^. In addition, FZHY completed phase II clinical trials in HCV patients with hepatic fibrosis in USA (Trial No. NCT00854087). It was reported that patients receiving FZHY alleviated liver fibrosis^21^. In the present study, we examined the effects of FZHY on experimental sinusoidal capillarization and liver angiogenesis.

## Materials and Methods

### Drugs

FZHY recipe, composing 4 g Salvia, 2 g Peach kernel, 8 g Fermented Cordycepssinensis, 2 g Pine pollen, 6 g Gynostemma and 2 g Schisandra, was purchased from Shanghai Sundise Traditional Chinese Medicine Co., Ltd. SORA was purchased from Bayer.

### Animal models

Six-week old male C57BL/6 mice were obtained from Beijing Vital River Laboratory Animal Technology Co., Ltd (Beijing, China). Forty-eight mice were randomly assigned to four groups (n = 12 each) receiving double-distilled water (DDW), carbon tetrachloride (CCl_4_), CCl_4_ + SORAand CCl_4_ + FZHY treatment, respectively. For CCl_4_-treated mice, animals were injected with 2 ml/kg body weight CCl_4_ (15% v/v in olive oil) intraperitoneally for 6 weeks, three times each week. Administration of FZHY recipe (4.8 g/kg body weight) or SORA (4 mg/kg body weight) was performed by intragastric gavagedaily since the fourth week after CCl_4_ challenge.

In addition, five-week old male ICR mice (experimental Animal Centre, Chinese Academy of Sciences, Shanghai, China) were used for dimethylnitrosamine (DMN) model. Fourty-eight mice were randomly assigned to four groups (n = 12 each) receiving DDW, DMN, DMN + SORA and DMN + FZHY, respectively. Mice received intraperitoneally injection of 5 mg/kg DMN (Tokyo Chemical Industry Co Ltd, Tokyo, Japan) for 3 weeks, three times a week. Administration of FZHY recipe (4.8 g/kg body weight) or SORA (4 mg/kg body weight) was performed by intragastric gavage (ig) daily starting from the fourth week

Animals were sacrificed 24 hr after the last administration. Livers and serum were collected for histological, cytological, biochemical, and molecular analyses. All animal experiments were approved by the institutional animal ethics committees of the Laboratory Animal Center at Shanghai University of Traditional Chinese Medicine, Shanghai, China (Ethics Number: SZY2013036, SZY201508006). All protocols and experimental procedures were conducted in accordance with the relevant institutional guidelines and regulations.

### Histological examinations

Collected liver tissues were fixed in 4% formalin and embedded in paraffin. Sections (4 μm) were stained with hematoxylin & eosin (H&E) and Sirius red staining. A grading system previously described was used to evaluate stages of fibrosis^22^.

### Immunohistochemistry

Paraffin-embedded slices (4 μm) were used for immunohistochemical staining. Endogenous peroxidase activity was blocked by methanol with 3% H_2_O_2_ and bovine serum albumin. After washing with PBS, slices were incubated with primary antibody (Supplementary Table 1) at 4 °C overnight. At the second day, slices were incubated with horseradish peroxidase (HRP)-conjugated secondary antibody for 1 h at 37 °C. Diaminobenzidine (DAB) was used as a chromogen followed by hematoxylin counterstaining.

### Immunofluorescent staining

Collected liver tissues were put into Tissue-Tek OCT embedding medium and snap-frozen in liquid nitrogen. Then, tissues were fixed with acetone for 10 min, washed with PBS, and blocked with 0.5% bovine serum albumin for 1 h at 37 °C. Tissues were incubated with primary antibodiesat 4 °C overnight. Next day, fluorescein isothiocyanate-labeled secondary antibodies were added to the samples for 1 h at 37 °C. Subsequently, Hoechst was incubated with the samples for 5 min. The tissues were observed under confocal microscopy for imaging.

### Analysis with planar X-ray in-line phase-contrast imaging (ILPCI)

The whole liver lobes were fixed with 4% PFA for 72 h. Then, the samples were subject to sequential alcohol dehydration followed by desiccant drying. The measurement was performed by beam line (BL13W1, Shanghai Synchrotron Radiation Facility, Shanghai). The tunable energy range of used X-ray beam was 9–65 keV. The pixel size of the detector was 3.7 μm. The field of view was 49.8 mm (H) × 5.04 mm (V). The detector was positioned at 120 cm from the sample. The imaging was obtained by 15 keV using a perfect silicon crystal.

### Cells

Human hepatic sinusoidal endothelial cell line (HHSEC) was purchased from Scien Cell Research Laboratories (San Diego, California). Cells were cultured in endothelial cell complete medium supplemented with 5% fetal bovine serum (FBS) were incubated at 37 °C and 5% CO_2_. Medium was changed every 2 days.

Human umbilical vein endothelial cell (HUVEC) was purchased from the American Type Culture Collection (ATCC) (Manassas, VA, USA). Cells were cultured in dulbecco’s modified eagle medium (DMEM) with 5% FBS.

### Toxicity assay

Toxicity tests were applied to HHSEC cells treated with FZHY recipe with different concentrations (62.5/4, 62.5/2, 62.5, 125, 250, 500 and 1000 μg/ml) for 48 hrs. The proliferation of cells was induced with serum-free 2% (V/V) endothelial cell growth supplements (ECGS, Science Research Laboratories) for 48 h.

### Cell proliferation assay

EDU-DNA incorporation assay was used to measure cell proliferation. Following different treatments, cells were incubated with ECM containing EDU for 4 h. Incorporated EDU was detected and visualized by Apollos 643 staining. Images were taken by Cellomics Array Scan VTI HCS Reader.

### Matrigel HHSEC tube formation assay

HHSEC cells cultured in 48-well plates coated with Growth Factor-Reduced Matrigel (BD Biosciences, Oxford, UK) were polymerized in an incubator at 37 °C for 9 h. Tube formation was visualized under Olympus DP71 Research stereomicroscope and analyzed by counting tube number using software ImageJ.

### Angiogenesis assay in fibrin gel

Cytodex 3 microcarrier beads (Amersham Pharmacia Biotech, Piscataway, NJ) were coated with HUVEC (400 HUVEC per bead). The coated beads suspensions were cultured in a siliconized glass bottle (Sigmacote) overnight. Next day, washed the coated beads twice with PBS. Then, 100 beads/ml coated beads were suspended in 2.0 mg/ml fibrinogen solution. The 24-well plates were pipetted gently by 500 μl fibrinogen/bead solution composing of 0.625 Units/ml thrombin and 0.15 Units/ml aprotinin four to five times. The fibrinogen/bead solution was clotted for 5 min at room temperature and then was putted at an incubator with 37 °C and 5% CO_2_ for 15 min. Complete HUVEC media containing 10% FBS was added to the embedded fibrin gel to inactivate thrombin overreaction. After 4 hours of culture, cells received different treatment as planned. Angiogenesis was visualized by microscopy at different time points.

### Scanning electron microscopy (SEM)

The livers were perfused with physiological saline via the portal vein and fixed with 2.5% glutaraldehyde. Collected tissues were cut into small pieces and fixed in 4% osmium for 1 h. Then, the tissues were processed for sequential alcohol dehydration and were infiltrated with t-butyl alcohol. After freezing, the tissues were vacuum-dried and then coated with ion sputter Hitachi E-1030 (Hitachi, Tokyo, Japan) for analysis with the scanning electron microscope SEMS-4100 (Hitachi).

The HHSEC cells were plated on collagen-coated cell culture inserts (BD Biosciences, Bedford, MA) and processed with the same protocols described above.

### Transmission electron microscopy (TEM)

Tissue samples were immersed in acetone and carefully dissected into pertinent regions of interest from the anterior apex, kept with one dimension of the tissue at 1~3 mm. After 15 min incubation in acetone, tissues were putted in mixture of acetone and 618 resin with increasing concentration gradients. Then, specimens were oriented and positioned in labeled molds and placed in an oven for curing. The hardened blocks were trimmed and prepared for 1.0–1.5 μm semi-thin sections. Sections were mounted on microscope slides and stained with lead citrate for 1 min at 80 °C. The stained ultrathin sections were observed with a transmission electron microscope.

### Western blot

Liver or HHSEC lysates were separated on 10% SDS-PAGE gels and transferred into nitrocellulose membranes. The membranes were blocked with 5% BSA and incubated with different primary antibodies at 4 °C overnight. After three times of washing with PBST, membranes were incubated with secondary antibodies (Supplementary Table 1) at room temperature for 1 hr. After additional three washing, membranes were scanned and imaged with Li-Cor odyssey.

### Quantitative real-time PCR

Total RNA was extracted from frozen mouse liver tissues using a kit according to the manufacturer’s instructions. The reaction conditions for PCR were: denaturation for 5 min at 94 °C, annealing for 1 min at 60 °C, and elongation for 1.5 min at 72 °C. Primersequences were shown in Supplementary Table 2.

### Statistical analyses

The one-way analysis of variance (ANOVA) was performed to calculate normally distributed continuous variables between the different groups. The data are presented as mean ± standard error of the mean (SEM). The differences were considered as significant when p < 0.05. The Results of ANOVA were shown in Supplementary Table 3.

## Results

### FZHY recipe ameliorated CCl_4_-induced liver injury in mice

**Figure 1:**
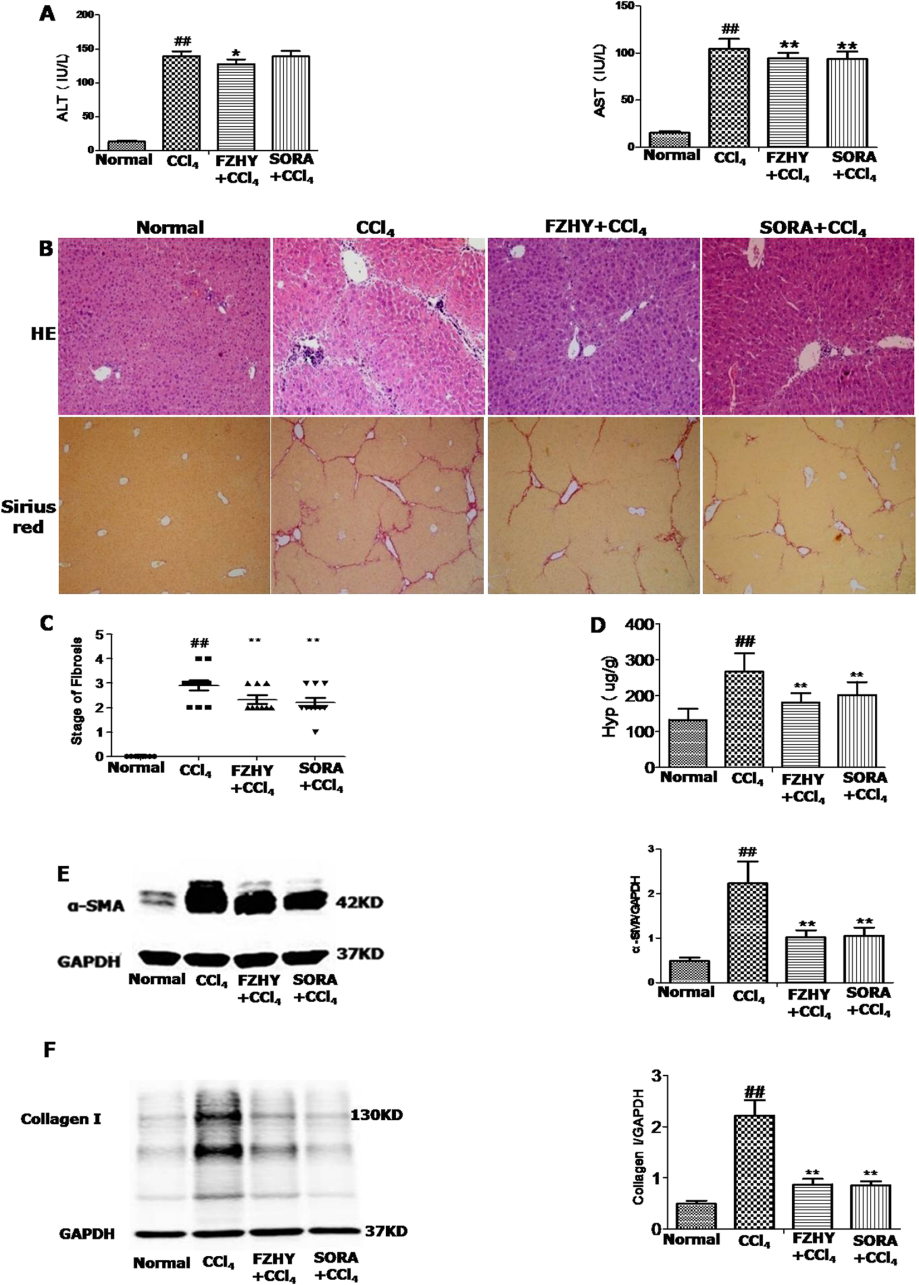
FZHY recipe ameliorated CCl_4_-induced liver injury in mice. (**A**) ALT and AST levels were measured in mice with different treatment. (**B**) Inflammatory and fibrotic degree were examined by hematoxylin and eosin (H&E) and Sirius red staining (original magnification ×100). (**C**) Sirius red positivecollagen areas were quantified. (**D**) Liver Hydroxyproline content was measured byJamall’s method^31^. (**E**,**F**) Protein expression of α-SMA and collagen type I in mice livers was measured by Western blot. ^#^And *P < 0.05, ^##^and **P < 0.01.

Firstly, we assessed the effects of FZHY in mice that were administered with CCl_4_ for 6 weeks. Six weeks of CCl_4_ administration in the mice induced high levels of serum ALT and AST, which were partly reduced by FZHY treatment. SORA decreased CCl_4_-induced levels of serum AST, but not ALT (Fig. 1A). Consistent with liver function parameters, HE staining revealed reduced inflammatory degree in both FZHY-and SORA-treated mice compared with the CCl_4_-administrated mice without treatment. Subsequently, the degree of fibrosis in mice with different administrations was assessed with Sirius red staining (Fig. 1B,C), content of α-SMA, collagen I and hydroxyproline (Fig. 1D–F). All measurements showed that CCl_4_-induced liver fibrosis indices in the mice were significantly inhibited by either FZHY or SORA treatment^22,23^.

### FZHY recipe ameliorated CCl_4_-inducedangiogenesis and sinusoidal capillarization

**Figure 2:**
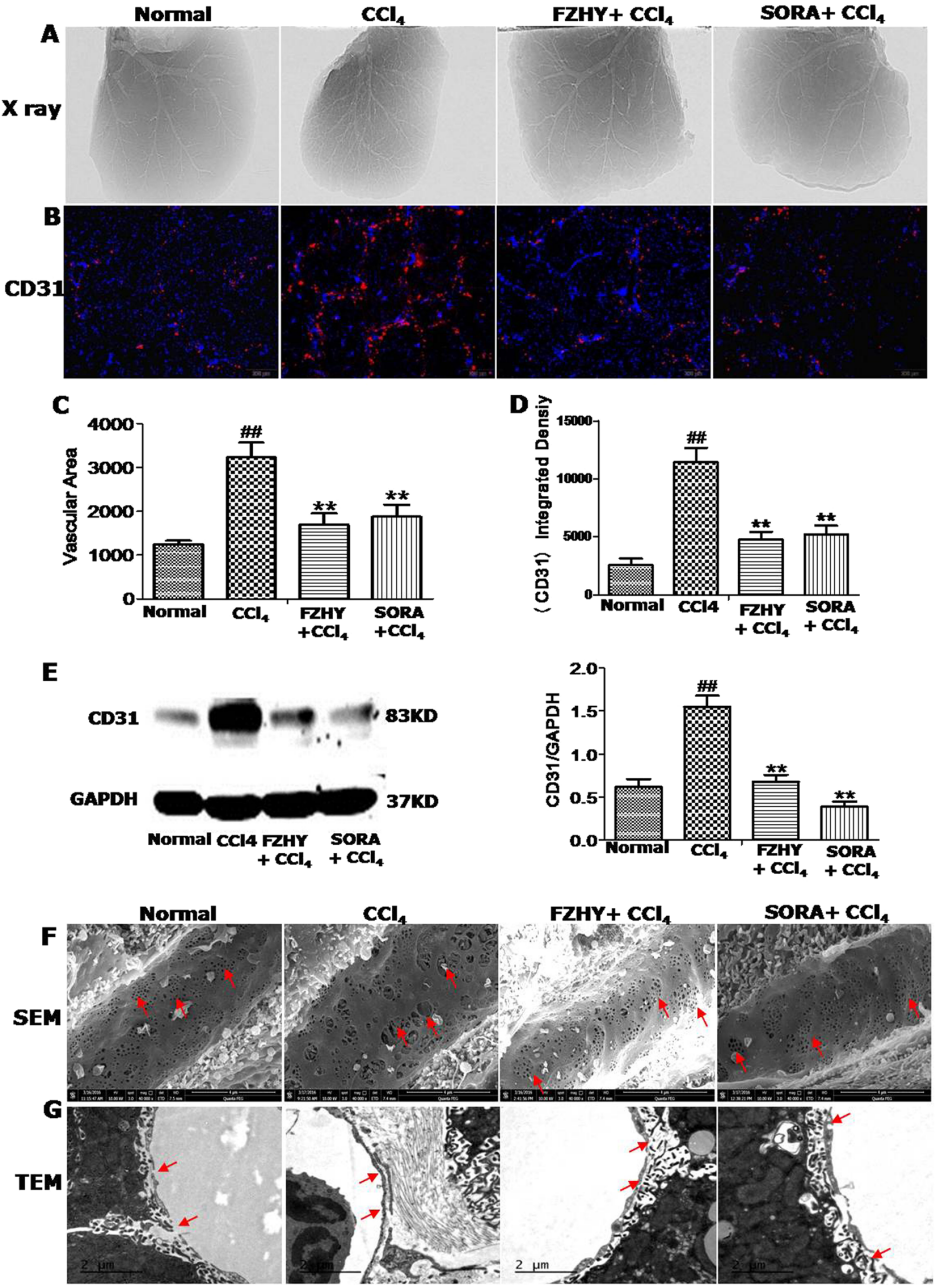
FZHY recipe ameliorated CCl_4_-induced angiogenesis and sinusoidal capillarization. (**A**,**C**) Remodeling of liver vessel was measured by ILPCI-CT in mice with different treatments as indicated. (**B**,**D**,**E**) CD31 expression was examined and quantified by immunofluorescence and western blot. (**F**,**G**) Liver sinusoidal structural changes were observed by scanning electron microscope (SEM) and transmission electron microscope (TEM). ^#^And *P < 0.05, ^##^and **P < 0.01.

Next, we investigated the effects of FZHY on CCl_4_-induced liver angiogenesis in mice. Measurement with ILPCI-CT showed that like SORA, FZHY significantly decreased the angiogenesis in mouse livers induced by 6 weeks of CCl_4_ treatment, although the inhibitory capacity of FZHY 4 g/kg was weaker than 4 mg/kg of SORA (Fig. 2A,C).

In addition to evaluating the LSECs phenotype changes, we examined the expression of CD31 with immunofluorescence staining and western blot. CCl_4_-induced CD31 expression was markedly inhibited by either SORA or FZHY treatment (Fig. 2B,D,E).

Further, we examined the effects of FZHY on CCl_4_-induced sinusoidal capillarization with TEM and SEM. Both TEM and SEM demonstrated that 6 weeks of CCl_4_ administration remarkably decreased the number and diameters of fenestra of liver sinusoidal endothelial cells (LSEC) in the mice (Fig. 2F). The sinusoidal capillarization was significantly inhibited by FZHY or SORA treatment (Fig. 2G).

### FZHY recipe ameliorated DMN-induced liver fibrosis, angiogenesis and sinusoidal capillarization

**Figure 3:**
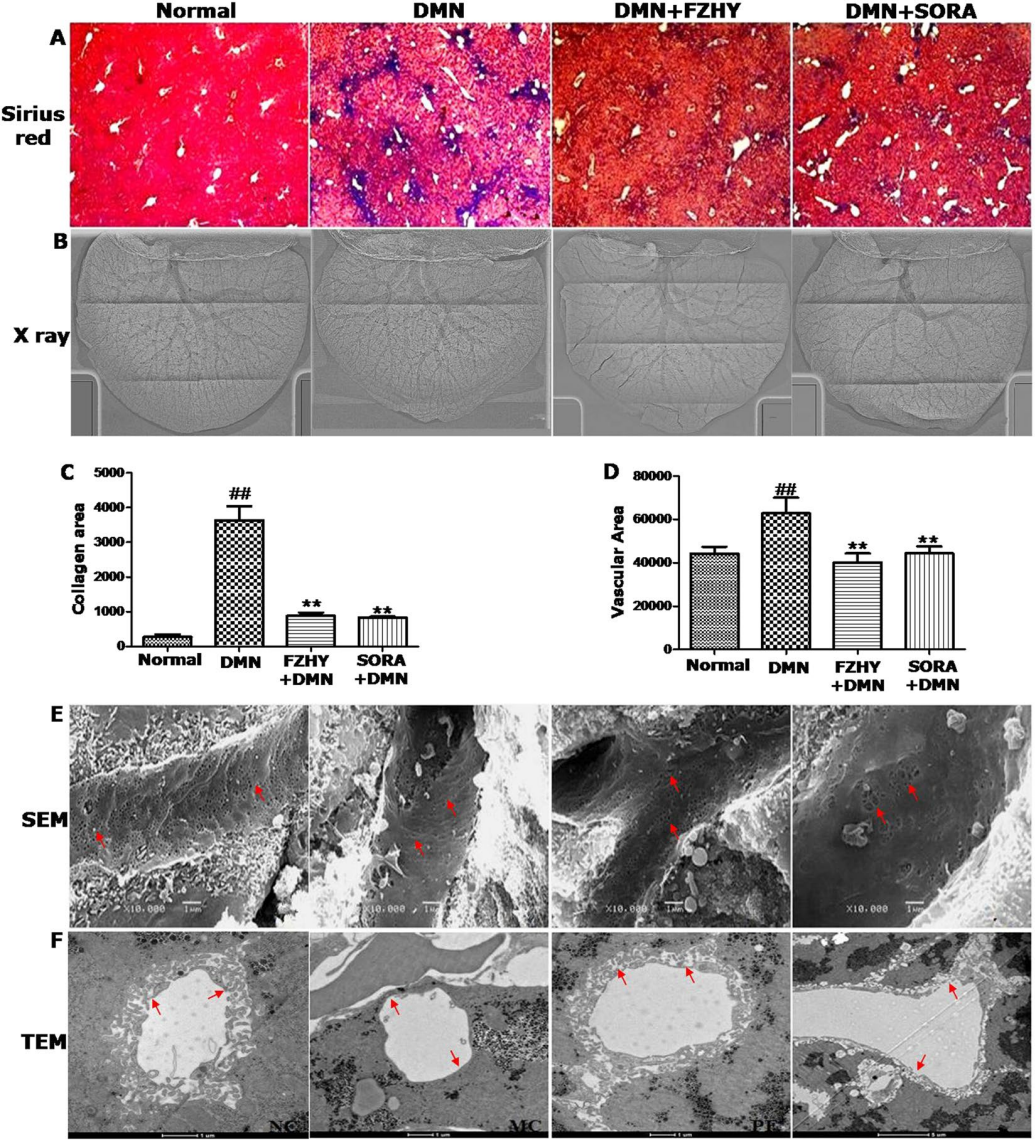
FZHY recipe ameliorated DMN-induced liver fibrosis, angiogenesis and sinusoidal capillarization. (**A**,**C**) Collagen deposition visualized in Sirius red and Masson staining (original magnification ×100). (**B**,**D**) Remodeling of liver vessel was measured by ILPCI-CT in mice with different administrations. (**E**,**F**) Liver sinusoidal structural changes were observed by scanning electron microscope (SEM) and transmission electron microscope (TEM).

To further verify the effects of FZHY on DMN-induced liver angiogenesis in mice, we confirmed that DMN administration induced collagen deposition in liver tissue, as assessed with Sirius red staining. Collagen deposition was obviously ameliorated in the SORA and FZHY-treated groups (Fig. 3A,C).

Next, Measurement with ILPCI-CT showed that like SORA, FZHY decreased DMN-induced angiogenesis in mouse livers (Fig. 3B,D). Both TEM and SEM demonstrated that DMN administration remarkably decreased the number and diameters of fenestra of liver sinusoidal endothelial cells (LSEC) in the mice. The sinusoidal capillarization was significantly inhibited by FZHY or SORA treatment (Fig. 3E,F).

### FZHY recipe reduced expression of angiogenesis-associated factors

**Figure 4:**
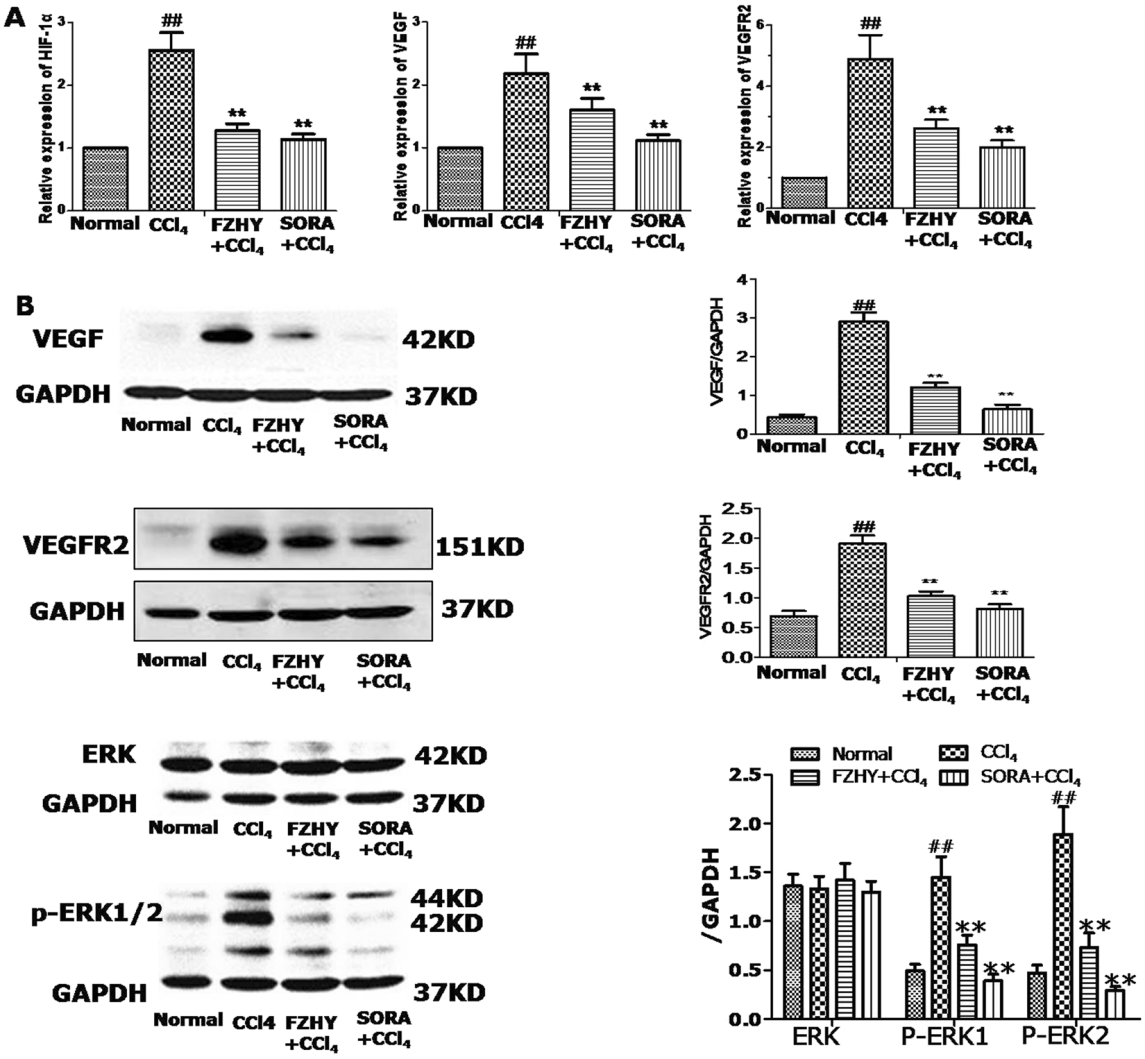
FZHY recipe reduced angiogenesis-related factors. (**A**) qRT-PCR was performed to analyze mRNA expression of HIF-1α, VEGF and VEGF-R2in mouse liver tissues. (**B**) Western Blot was used to examine protein expression of VEGF, VEGFR2, ERK1/2 and phosphorylated ERK1/2. GAPDH was used as loading control. The experiments were repeated at least three times. Data are presented as means ± SEM (**B**,**D**). ^#^And *P < 0.05, ^##^and **P < 0.01.

To investigate how FZHY impacts angiogenesis in mice, we measured the impact of the compound on the expression of angiogenesis-associated factors. CCl_4_ administration in the mice induced mRNA expression ofHIF-1α, VEGF and VEGF-R2, which were partly reduced by FZHY or SORA treatment (Fig. 4A). Western blot further confirmed that FZHY or SORA also inhibited protein expression of VEGF and VEGF-R2 in CCl_4_-administrated mice. In addition, FZHY or SORA treatment decreasedCCl_4_-induced p-ERK1/2 levels (Fig. 4B).

### FZHY recipe inhibited VEGF-dependent HHSEC proliferation and tube formation

**Figure 5:**
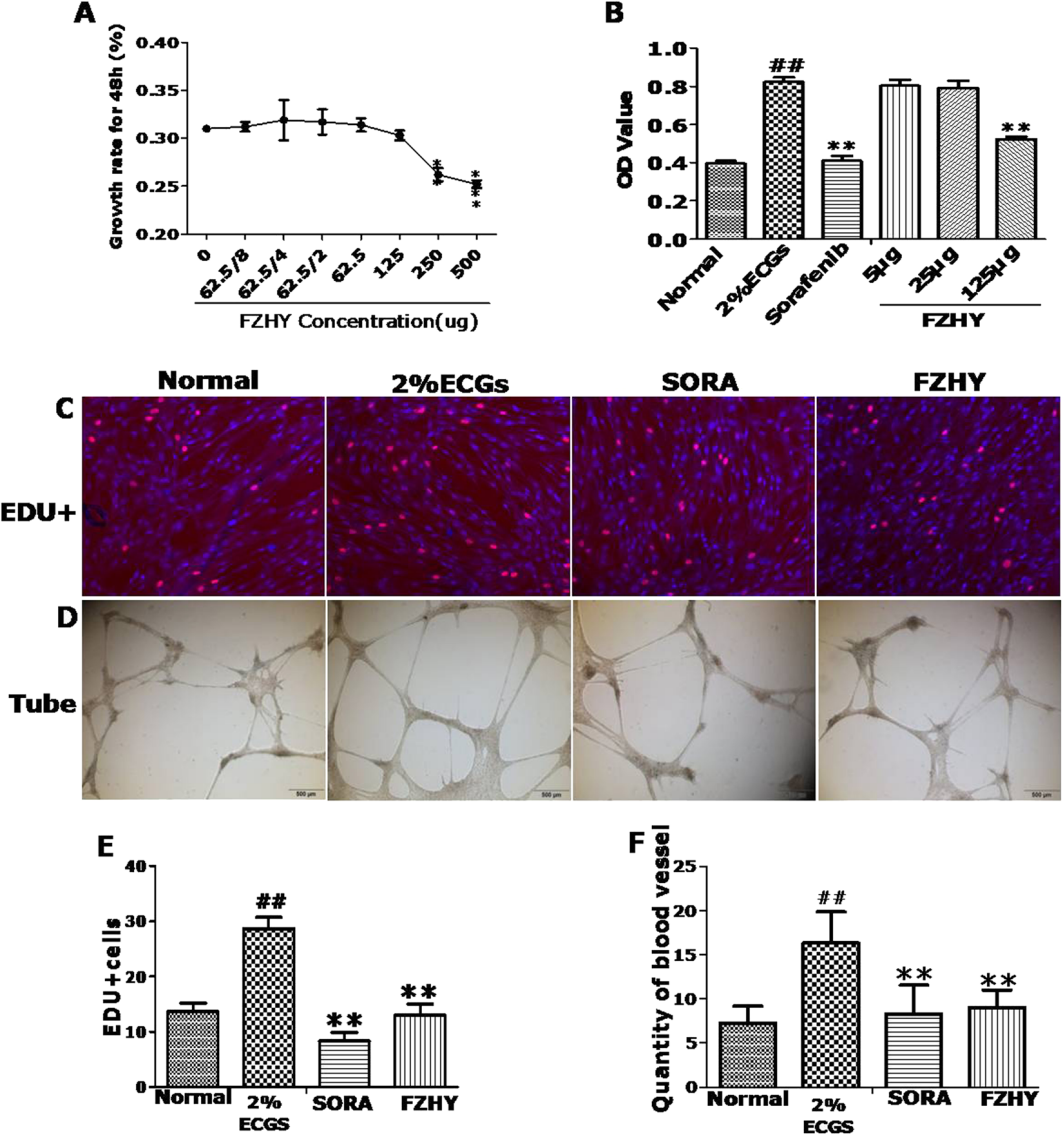
FZHY recipe inhibited VEGF-induced proliferation and tube formation of HHSEC cells. (**A**,**B**) Growth and vitality of HHSEC cells was assayed by CCK8 assay. (**C**) Cell proliferation was analyzed by EDU assay. (**D**) Matrigel tube formation was measured for three times. ^#^*P* < 0.05, ^##^*P* < 0.01; *P < 0.05, **P < 0.01.

To further assess the effects of FZHY on angiogenesis, we observed the impact of FZHY on HHSEC *in vitro*. Firstly, the toxicity of FZHY for HHSEC cells was assessed. FZHY did not have toxicity for the cells with dosage up to 125 mg/ml. Then, HHSEC cells were stimulated with VEGF for 48 h. VEGF-induced vitality and proliferation of HHSEC cells was significantly inhibited by 125 μg/ml of FZHY (Fig. 5A–C,E). On Matrigel cultured HHSEC cells, similar to SORA, FHZY exhibited a substantial inhibitory function for the formation of tube-like cellular networks (Fig. 5D,F).

### FZHY maintained the number of LSEC fenestra induced by VEGF, but decreased VEGF-induced LSEC protrusion

**Figure 6:**
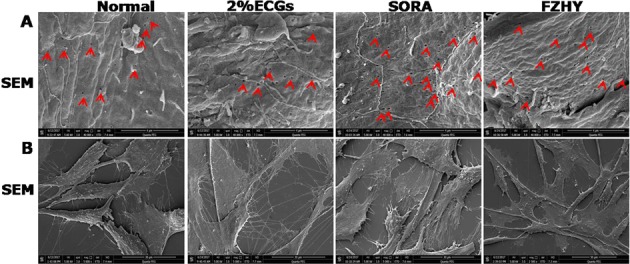
FZHY maintained the number of LSEC fenestra induced by VEGF, but decreased VEGF-induced LSEC protrusion. Sinusoidal structural changes of HHSECs were observed by scanning electron microscope (SEM).

Next, we performed SEM to observe morphological alteration of HHSEC cells upon FZHY treatment. VEGF administration increased the number of HHSEC fenestra, which were not impacted by either SORA or FZHY treatment (Fig. 6A). However, FZHY and SORA administration significantly decreased the number of HHSEC cell protrusions induced by VEGF (Fig. 6B).

### FZHY directly suppressed VEGF expression and downstream ERK phosphorylation

**Figure 7:**
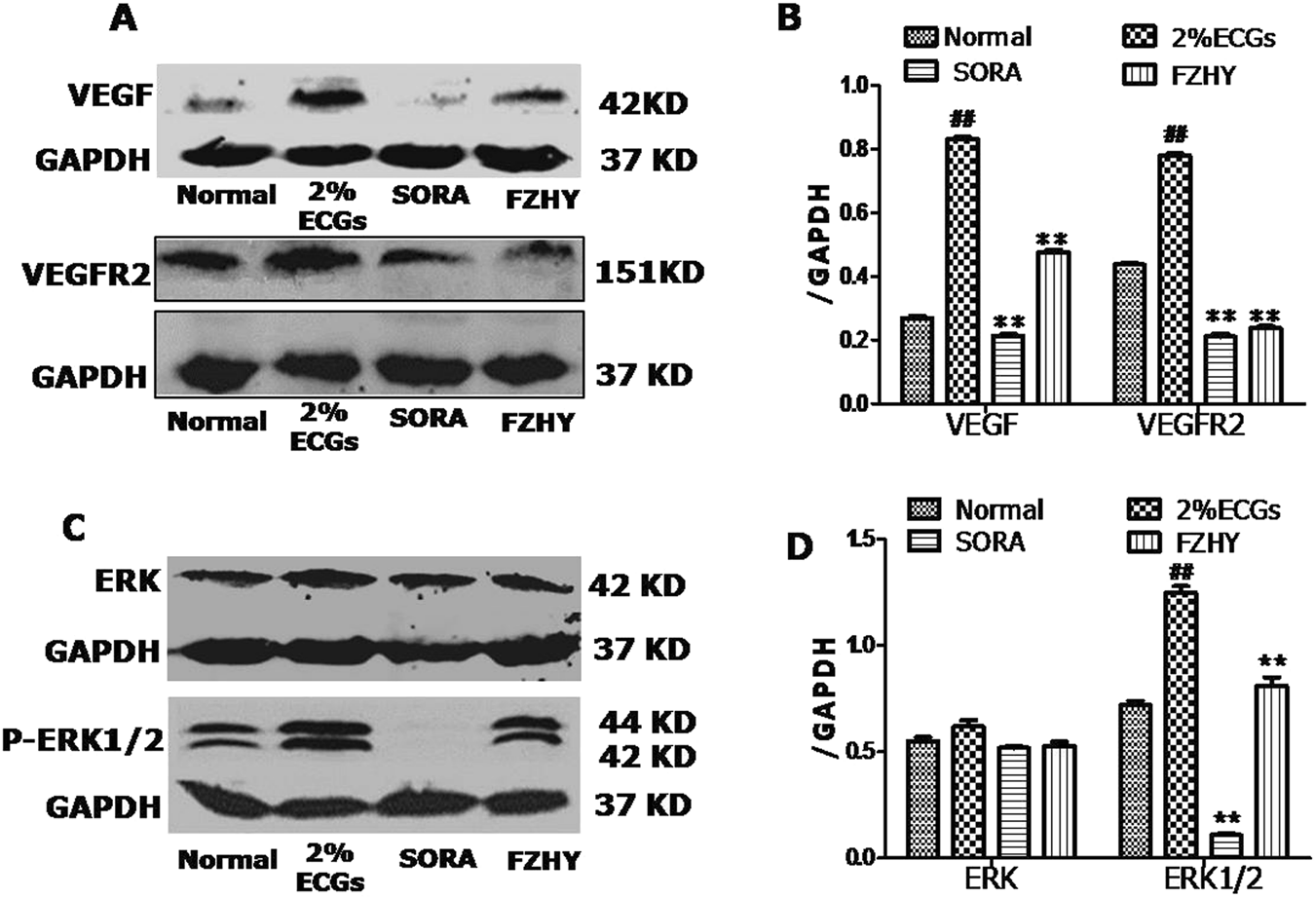
FZHY recipe suppressed angiogenesis via inhibiting VEGF signaling *in vitro*. Western blot for VEGF and VEGFR2 (**A**) phosphorylated and total ERK1/2 expression (**C**) are shown. GAPDH was used as loading control. The experiments were repeated at least three times. Data are presented as means ± SEM (**B**,**D**). ^#^And *P < 0.05, ^##^and **P < 0.01.

To elucidate how FZHY impacts the effects of VEGF, we examined expression of VEGF and its downstream ERK protein activation in HHSEC cells. Western blot analyses showed that FZHY suppressed VEGF, and p-ERK1/2 expression was increased in 2% ECGs, but minimal changes of ERK were observed. With FZHY or SORA treatment, VEGF and *p*-ERK1/2 expression were decreased, particularly down-regulated in SORA group, but minimal changes of ERK were observed (Fig. 7A–D).

### FZHY recipe inhibited angiogenesis development in 3D fibrin gel

**Figure 8:**
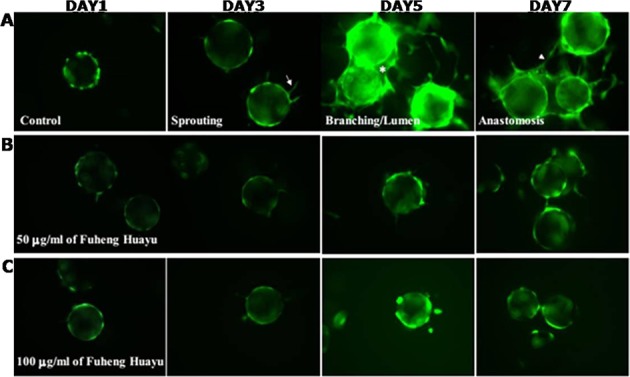
FZHY recipe inhibited angiogenesis in 3D Fibrin Gel. Sprouting, branching/lumen formation, and anastomosis were visualized in HUVECs with or without FZHY treatment.

In order to evaluate the effects of FZHY on angiogenesis, we performed sprouting assays in three dimensional (3D) cultured HUVEC cells. In untreated cells, HUVEC sprouting appeared at day-3 of culture. At day-5 and day-7 of culture, sprouting and anastomosis were robust (Fig. 8A). 50 μg/ml of FZHY markedly inhibited the sprouting of HUVEC cells, with only at day-7 of culture, some cells demonstrating short protrusion (Fig. 8B). 100 μg/ml of FZHY completely inhibited HUVEC migration and sprouting (Fig. 8C).

## Discussion

FZHY is a traditional Chinese medicinal formulation which has successfully treated patients with HBV-related liver fibrosis and mice with different models of liver fibrosis^14,20,24,25^. Recently, a phase II clinical trial performed in the USA also appeared to show that the compound benefited patients with HCV-associated liver fibrosis. The results from the current study show that anti-fibrotic effects of FZHY is reproducible. In both CCl_4_ and DMN-induced liver injury models, FZHY remarkably alleviated liver fibrosis, inflammation and hepatocyte death, indicating a convincing therapeutic effect of FZHY in treating liver fibrosis.

It is well recognized that activation of HSC induces de-differentiation of LSEC, characterized by loss of fenestra, sinusoidal capillarization^23,26^. Pathological angiogenesis is commonly observed in advanced fibrosis^27^. Inflammation-associated angiogenesis might contribute to the initiation of liver fibrosis, and progress to cirrhosis and HCC^11,28^. In the current study, we found that FZHY significantly inhibited expression of α-SMA, a marker of HSC activation^29^. This finding led us to hypothesize that FZHY has a role of inhibiting sinusoidal capillarization. As expected, electronic microscopy confirmed that CCl_4_- or DMN-induced fenestra loss of LSEC were recovered following FZHY or SORA treatment. *In vitro*, we further examined the effects of FZHY on fenestra of HHSEC cells. Consistent with animal models, FZHY treatment maintained the existence of fenestra of HHSECs.

Besides inhibiting sinusoidal capillarization, FZHY shows impressive effects on liver angiogenesis. Planar X-ray in-line phase-contrast imaging analysis clearly showed that liver angiogenesis induced by CCl_4_ or DMN administration was inhibited by FZHY treatment. In line with the animal models, *in vitro* studies based on HHSEC and HUVEC 3D fibril gel models further demonstrated that FZHY was capable of inhibiting VEGF-induced angiogenesis of LSEC and other endothelial cells.

Vessel formation was associated with strong expression of the pivotal proangiogenic growth factor VEGF and its receptor VEGFR2, which have been earlier considered a prerequisite for fibrogenesis *in vivo*^1,30^. In this study, we also assessed how FZHY impacted liver angiogenesis. We found that FZHY can inhibit expression of VEGF, VEGFR2 and activation of VEGF downstream substrates, e.g. ERK, *in vivo* and *in vitro*. This finding suggests that the effects of FZHY on blocking sinusoidal capillarization and liver angiogenesis are not only through inhibiting activation of HSC, but also via directly inhibiting VEGF signaling.

Taken together, given no available approaches to inhibit sinusoidal capillarization and liver angiogenesis, impressive effects of FZHY on the two key events of liver fibrosis progressing to cirrhosis shed a light on treatment of liver cirrhosis. Of course, the results based on *in vitro* and animal models cannot reflect patient’s situation in a large context. Species, etiology, natural histology and distinct pathophysiological response between patients and animals make good experimental achievements cannot translate into clinical success. In the future, a clinical trial should be considered to assess the role of FZHY on liver cirrhosis.

## Supplementary information

supplementary materials
