# Supplementary material for: Fuzhenghuayu Decoction ameliorates hepatic fibrosis by attenuating experimental sinusoidal capillarization and liver angiogenesis
Source: Sci Rep. 2019 Dec 10;9:18719. doi: 10.1038/s41598-019-54663-4 (PMC6904731; doi:10.1038/s41598-019-54663-4)

**Fuzhenghuayu Decoction ameliorates hepatic fibrosis by attenuating experimental** **sinusoidal capillarization and liver angiogenesis**

Hong-liang Liu^1,2,3^*, Jing Lv^1,2,3^*, Zhi-min Zhao^1,2^, An-ming Xiong^4^, Ye Tan^1^, Jeffrey S. Glenn^4^,Yan-yan Tao^1^, Hong-lei Weng^5^  and Cheng-hai Liu^1,2,3^*

**Supplementary Table 1. Primary antibodies**

| Antibody | Isotype | Suppliers | Cat. no. | Dilution |
| --- | --- | --- | --- | --- |
| α-SMA | Rabbit polyclonal IgG | Abcam | ab5694 | 1:500 |
| Collagen Type I | Mice IgG1 | Sigma | C2456 | 1:200 |
| CD31 | Goat polyclonal IgG | Abcam | ab24590 | 1:200 |
| VEGF | Rabbit polyclonal IgG | Abcam | ab46154 | 1:200 |
| VEGF-R2 | Rabbit polyclonal IgG | Abcam | Ab39638 | 1:400 |
| GAPDH | Mice Monoclonal | KangChen | KC-5G4 | 1:10000 |
| ERK  P-ERK  Collagen Type IV | Mouse IgG2b  Mouse IgG2a  Rabbit polyclonal IgG | Santa Cruz  Santa Cruz  Abcam | sc-1647  sc-7383  ab6586 | 1:200  1:200  1:200 |

**Fluorophore-conjugated secondary antibodies**

| Antibody | Isotype | Suppliers | Cat. no. | Dilution |
| --- | --- | --- | --- | --- |
| secondary antibodies | Goat anti-Rabbit | LI-COR | 926-68071 | 1:5000 |
| secondary antibodies | Goat anti-Mouse | LI-COR | 926-32210 | 1:5000 |

**Supplementary Table 2.Real-time quantitative PCR primers**

| Gene | Forward (5’→3’) | Reverse (5’→3’) |
| --- | --- | --- |
| HIF-1α | 5'-CCAGATTCAAGATCAGCCAGCA -3' | 5'-GCTGTCCACATCAAAGCAGTACTA-3' |
| VEGF | 5'-ACATTGGCTCACTTCCAGAAACAC-3' | 5'-GGTTGGAACCGGCATCTTTATC-3' |
| VEGF-R2 | 5'-GGGATGGTCCTTGCATCAGAA -3' | 5'-ACTGGTAGCCACTGGTCTGGTTG -3' |
| β-actin | 5'-TGACGAGGCCCAGAGCAAGA -3' | 5'-ATGGGCACAGTGTGGGTGAC -3' |

**Supplementary Table 3.** **The** **Results of ANOVA test**

|  | F Value | DOF | P Value | Partial Eta Squared |
| --- | --- | --- | --- | --- |
| Fig1A:ALT | 97.689 | 3 | ＜0.001 | 0.913 |
| Fig1A:AST | 44.035 | 3 | ＜0.001 | 0.825 |
| Fig1D:Hyp | 14.193 | 3 | ＜0.001 | 0.612 |
| Fig1E:α-SMA | 195.096 | 3 | ＜0.001 | 0.987 |
| Fig1F:Collgen Ⅰ | 173.251 | 3 | ＜0.001 | 0.985 |
| Fig2C:Vascular | 460.093 | 3 | ＜0.001 | 0.994 |
| Fig2D:CD31 | 2545.299 | 3 | ＜0.001 | 0.999 |
| Fig2E:CD31 | 316.379 | 3 | ＜0.001 | 0.992 |
| Fig3C:Collgen Ⅰ | 21209.32 | 3 | ＜0.001 | 1 |
| Fig3D:Vascular | 1222.739 | 3 | ＜0.001 | 0.998 |
| Fig4A:HIF-1α | 195.077 | 3 | ＜0.001 | 0.987 |
| Fig4A:VEGF | 42.878 | 3 | ＜0.001 | 0.941 |
| Fig4A:VEGFR2 | 162.418 | 3 | ＜0.001 | 0.984 |
| Fig4B:VEGF | 967.079 | 3 | ＜0.001 | 0.997 |
| Fig4B:VEGFR2 | 479.329 | 3 | ＜0.001 | 0.994 |
| Fig4B:ERK | 0.189 | 3 | 0.901 | 0.066 |
| Fig4B:p-ERK1 | 492.906 | 3 | ＜0.001 | 0.995 |
| Fig4B:p-ERK2 | 541.053 | 3 | ＜0.001 | 0.995 |
| Fig5E:EDU+ | 65.487 | 3 | ＜0.001 | 0.961 |
| Fig5F:Tube | 6.743 | 3 | 0.014 | 0.717 |
| Fig7B:VEGF | 658.637 | 3 | ＜0.001 | 0.922 |
| Fig7B:VEGFR2 | 210.382 | 3 | ＜0.001 | 0.987 |
| Fig7D:ERK | 0.27 | 3 | 0.845 | 0.092 |
| Fig7D:p-ERK1/2 | 801.378 | 3 | ＜0.001 | 0.997 |

**Original blots**

Figure.1E α-SMA


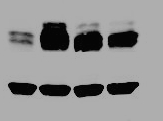


Figure.1F CollagenⅠ


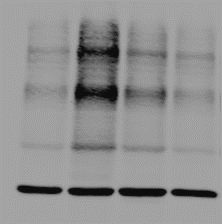


Figure.2E CD31


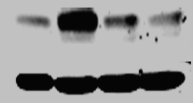


Figure.4B VEGF


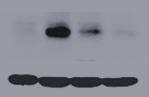


Figure.4B VEGFR2


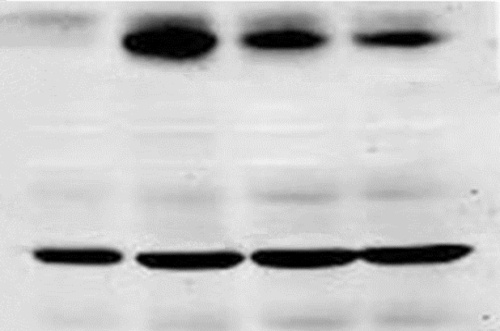


Figure.4B ERK


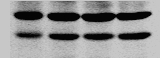


Figure.4B *p*-ERK1/2


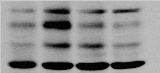


Figure.7A VEGF


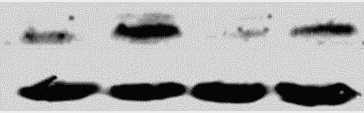


Figure.7A VEGFR2


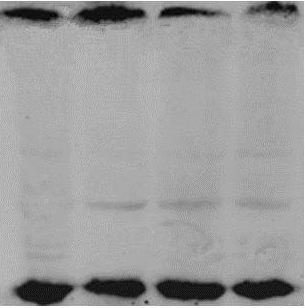


Figure.7C ERK


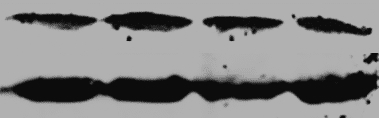


Figure.7C *p*-ERK1/2


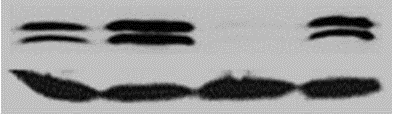

Supplement: Supplementary file 1 — supplementary materials [file 41598_2019_54663_MOESM1_ESM.docx]
